# Palliative Stereotactic Body Radiotherapy for Metastatic Clear Cell Sarcoma: A Case Report

**DOI:** 10.7759/cureus.114710

**Published:** 2026-08-18

**Authors:** Shinichiro Mizumatsu, Satoshi Tsukushi

**Affiliations:** 1 Department of Radiology, Narita Memorial Hospital, Toyohashi, JPN; 2 Department of Orthopedic Surgery and Sarcoma Center, Aichi Cancer Center Hospital, Nagoya, JPN

**Keywords:** clear cell sarcoma, every other day, flattening filter-free (fff), image-guided radiotherapy (igrt), linear accelerator (linac), oxray, palliative radiotherapy, radioresistant tumors, stereotactic body radiotherapy (sbrt), volumetric-modulated arc therapy (vmat)

## Abstract

Clear cell sarcoma (CCS) is an extremely rare malignancy. We report a case of metastatic CCS in which pain was successfully relieved with palliative stereotactic body radiotherapy (SBRT). The patient was a 57-year-old man diagnosed with CCS of the right heel five years prior. He had undergone seven surgical resections and chemotherapy (trabectedin and pazopanib) for the primary tumor and metastatic lesions. While receiving trabectedin therapy, he was referred to our facility for palliative radiotherapy. This was due to the rapid enlargement of a metastatic lesion in the right thigh. His symptom was pain in the right lower extremity. Metastatic lesions in the right thigh and right abdomen were targeted for SBRT. SBRT was delivered every other day using an O-ring linear accelerator (OXRAY) with 6-MV flattening filter-free beams, volumetric modulated arc therapy, and image-guided radiotherapy (IGRT) (thigh: treatment volume 319.57 mL, prescribed dose (the dose covering 95% of the target volume; D95) 30 Gy in 5 fractions, maximum dose 67.05 Gy, irradiation time 324 seconds/fraction, treatment duration 10 days; abdomen: treatment volume 36.2 mL, prescribed dose (D95) 36 Gy in 3 fractions, maximum dose 51.92 Gy, irradiation time 293 seconds/fraction, treatment duration five days). By the fifth day of SBRT, the pain in the right lower extremity completely resolved. Shrinkage of the right thigh metastasis was observed on cone-beam computed tomography (CT) scans of IGRT during the treatment period. CT imaging performed seven weeks after SBRT showed further shrinkage of both lesions. The pain did not recur until the patient died of renal failure two months after SBRT. No adverse events occurred during the clinical course. SBRT may be a viable palliative treatment option for CCS.

## Introduction

Clear cell sarcoma (CCS) is a highly malignant neoplasm that accounts for less than 1% of all soft tissue sarcomas, with an extremely low incidence rate of approximately one in a million [1]. It predominantly affects young adults in their 30s and 40s, with nearly half of cases arising in the deep soft tissues of the lower extremities, typically associated with tendons or aponeuroses [2-4]. While its cell of origin remains undetermined, the disease is characterized by a tendency for lymph node metastasis, a relatively rare occurrence among soft tissue sarcomas [3,5]. CCS is notoriously resistant to chemotherapy and conventional radiotherapy (CRT), making complete surgical resection the only modality for a cure [4-6]. The five-year survival rate is 47-67%, which drops to 20% if metastasis is present at diagnosis [5].

Stereotactic body radiotherapy (SBRT) delivers high doses of radiation precisely from multiple directions, offering potential efficacy even against tumors resistant to CRT [7]. SBRT utilizing flattening filter-free (FFF) beams [8], volumetric modulated arc therapy (VMAT) [9], and image-guided radiotherapy (IGRT) [10] represents a relatively new modality in radiation therapy. The advantages of this approach include reduced adverse events in surrounding tissues and enhanced therapeutic efficacy, achieved through improved delivery precision and dose conformity, as well as fewer treatment sessions and shorter irradiation times [11]. The OXRAY® system (Hitachi High-Tech Corporation, Tokyo, Japan) is a novel O-ring-type linear accelerator that entered clinical operation in June 2024 [12].

Here, we report a case in which SBRT using this system was administered to a metastatic lesion from CCS, resulting in a favorable palliative outcome.

## Case presentation

**Figure 1:**
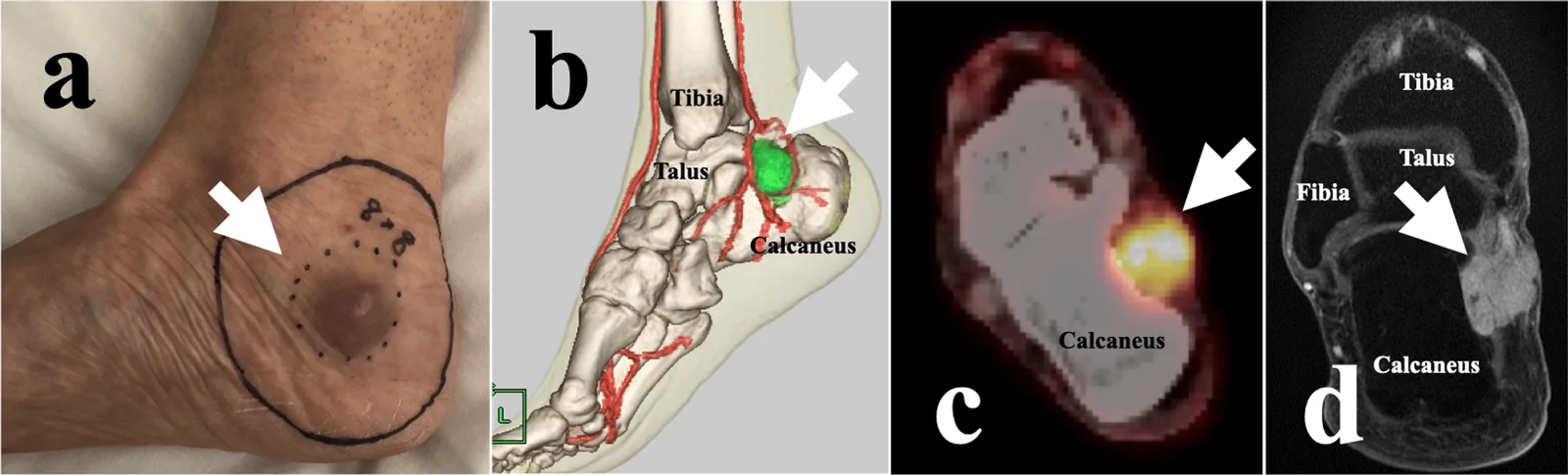
Preoperative lesion in the right heel (white arrows). (a) A photograph showing an elevated, circular, dark red lesion. (b) Sagittal 3D-CT showing the lesion above on the calcaneus. (c) Coronal PET/CT demonstrating intense FDG uptake in the lesion. (d) Coronal contrast-enhanced MRI demonstrating enhancement of the lesion. 3D, three-dimensional; CT, computed tomography; PET, positron emission tomography; FDG, 2-deoxy-2-[¹⁸F]fluoro-D-glucose; MRI, magnetic resonance imaging.

**Figure 2:**
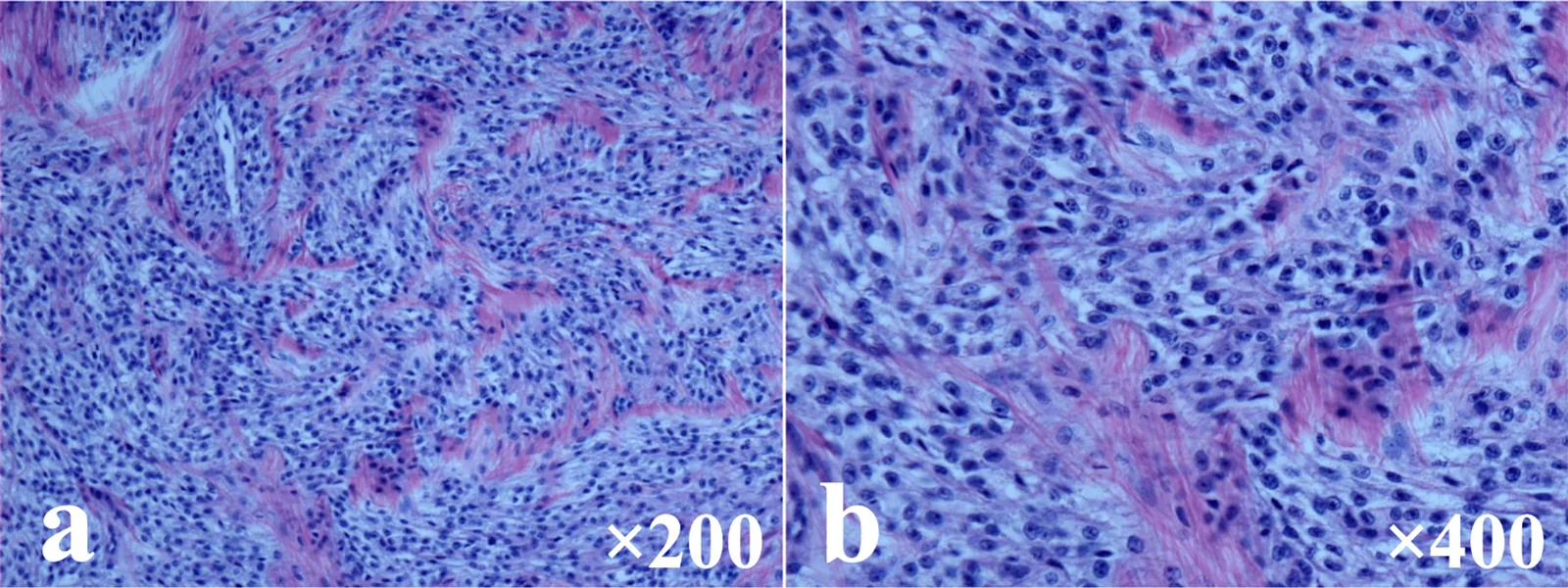
Histopathological findings (hematoxylin and eosin staining). (a) Low-power field (×200) and (b) high-power field (×400) of the nodular lesion in the right lower heel, which measured up to 23 × 20 mm. Atypical cells with clear cytoplasm proliferate in an alveolar pattern. Immunohistochemistry showed positivity for HMB-45, S-100, SOX-10, and MITF (focal), and negativity for CK OSCAR. The Ki-67 labeling index was approximately 30% at the tumor periphery. These findings led to a diagnosis of clear cell sarcoma. HMB-45, human melanoma black 45; SOX-10, SRY-box transcription factor 10; MITF, microphthalmia-associated transcription factor; CK, cytokeratin.

A 57-year-old male with CCS presented with a rapid enlargement of a metastatic lesion in the right thigh. Five years before presentation, the disease had manifested as a painless mass in his right heel (Figure 1), and a biopsy confirmed the diagnosis of CCS (Figure 2).

He underwent wide resection of the right heel tumor followed by free anterolateral thigh flap reconstruction. No postoperative chemotherapy was administered. Two years after the initial surgery, the cancer recurred and metastasized. Over the following years, he underwent a total of six surgical resections for both the primary tumor and metastatic lesions.

**Figure 3:**
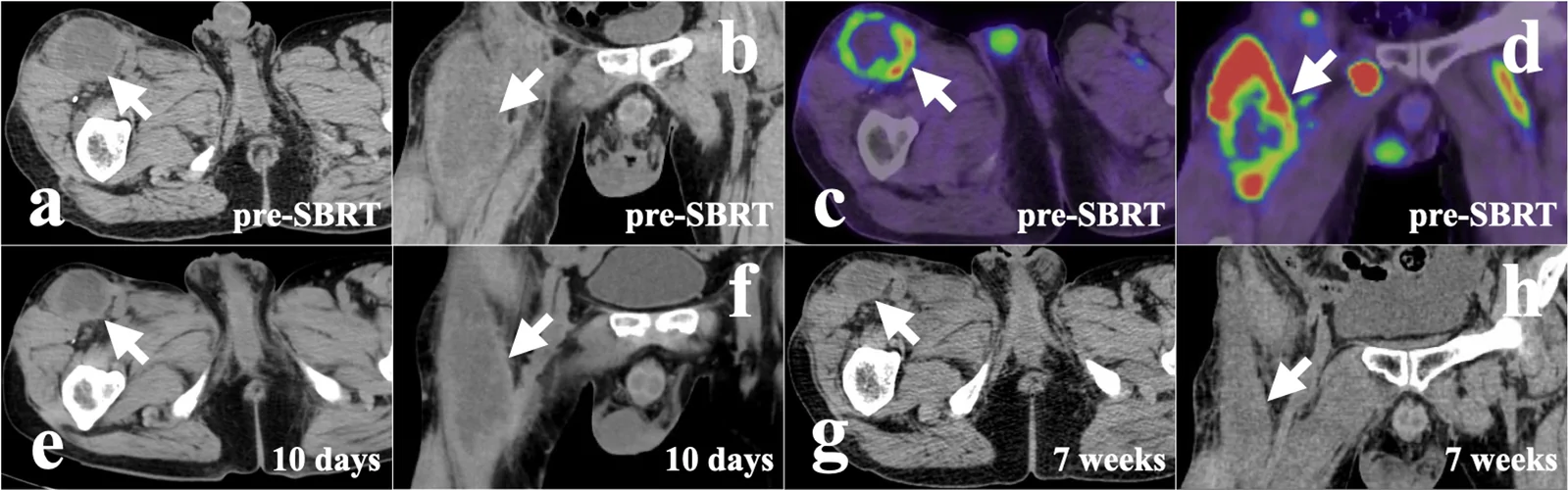
Metastatic lesion in the right thigh (white arrows). (a-d) Images obtained before SBRT. (a, b) Non-contrast CT showing the lesion with an isodense wall and internal low density. (c, d) PET/CT demonstrating intense FDG uptake in the wall of the lesion. (e, f) Non-contrast CT obtained 10 days after SBRT initiation (the final day of treatment), showing tumor regression. (g, h) Non-contrast CT obtained seven weeks after SBRT, showing further tumor regression. (a, c, e, g) Axial views and (b, d, f, h) coronal views. SBRT, stereotactic body radiotherapy; CT, computed tomography; PET, positron emission tomography; FDG, 2-deoxy-2-[¹⁸F]fluoro-D-glucose.

**Figure 4:**
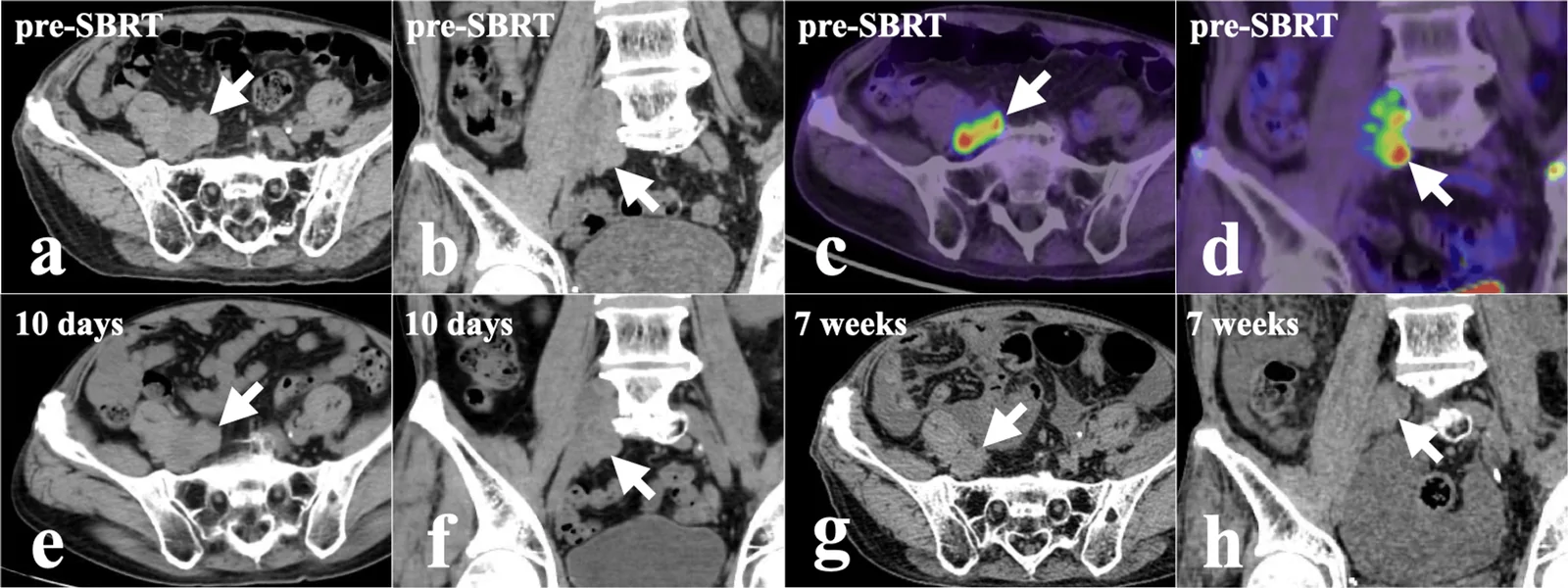
Metastatic lesion in the right abdomen (white arrows). (a-d) Images obtained before SBRT. (a, b) Non-contrast CT showing an isodense lesion. (c, d) PET/CT demonstrating intense FDG uptake in the lesion.  (e, f) Non-contrast CT obtained five days after SBRT initiation (the final day of treatment), showing tumor regression. (g, h) Non-contrast CT obtained seven weeks after SBRT, showing further tumor regression. (a, c, e, g) Axial views and (b, d, f, h) coronal views. SBRT, stereotactic body radiotherapy; CT, computed tomography; PET, positron emission tomography; FDG, 2-deoxy-2-[¹⁸F]fluoro-D-glucose.

Systemic therapy with trabectedin was initiated two years and 10 months after the initial surgery but was discontinued after five months due to a decline in cardiac function. He was subsequently switched to pazopanib; however, due to disease progression, trabectedin was resumed five months later. He was referred to our department for symptom palliation. At the initial visit, he was ambulatory, although his gait was unstable due to right lower-limb pain that worsened with extension. As the pain occurred only during movement, the patient did not take analgesics regularly. No skin breakdown or hemorrhage was observed in the right thigh. Computed tomography (CT), magnetic resonance imaging, and 2-deoxy-2-[^18^F] fluoro-D-glucose positron emission tomography/CT revealed multiple systemic metastases, including a soft tissue metastasis in the right thigh and a right abdominal metastasis (Figures 3a-3d; Figures 4a-4d).

**Figure 5:**
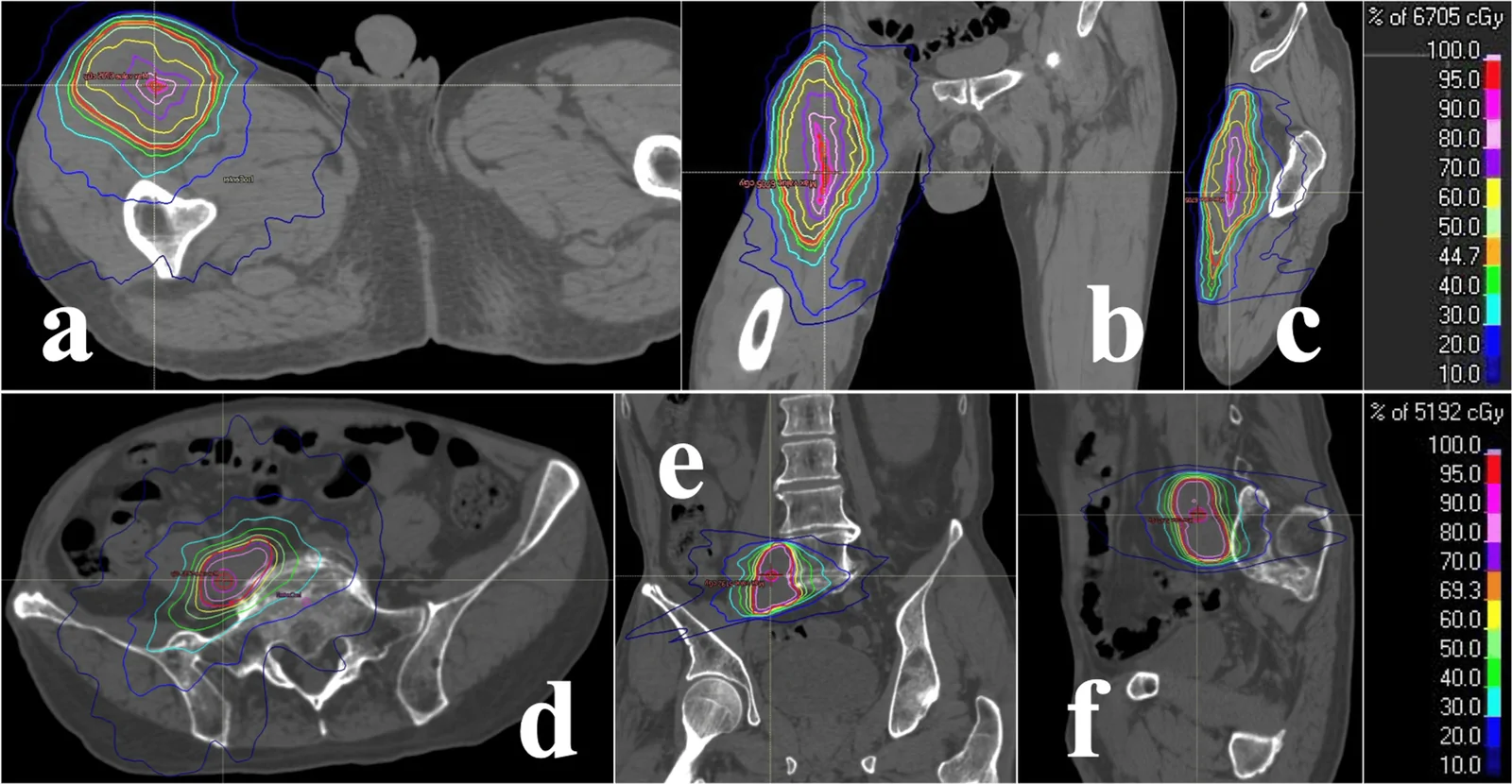
Planned dose distribution of SBRT. (a-c) Metastatic lesion in the right thigh. Isodose lines are shown relative to the maximum dose (100% = 67.05 Gy) on CT: (a) axial, (b) coronal, and (c) sagittal views. Irradiation time is 324 seconds per fraction via three arcs at O-ring angles of 0°, 15°, and 345° using the OXRAY system. (d-f) Metastatic lesion in the right abdomen. Isodose lines are shown relative to the maximum dose (100% = 51.92 Gy) on CT: (d) axial, (e) coronal, and (f) sagittal views. Irradiation time is 293 seconds per fraction via three arcs at O-ring angles of 0°, 20°, and 340° using the OXRAY system. SBRT, stereotactic body radiotherapy; CT, computed tomography.

The right thigh and the abdominal metastases were targeted for SBRT. The SBRT plan was generated using RayStation (RaySearch Laboratories, Stockholm, Sweden) (Figure 5).

SBRT was delivered using the OXRAY system with 6-MV FFF beams and VMAT. Treatment plans for both lesions utilized three arcs, with irradiation performed every other day. For the SBRT targeting the right thigh metastasis, the treatment parameters were as follows: treatment volume, 319.57 mL; prescribed dose (the dose covering 95% of the target volume; D95), 30 Gy in 5 fractions; maximum dose, 67.05 Gy; irradiation time, 324 seconds per fraction; and overall treatment duration, 10 days. For the right abdominal metastasis, the parameters were: treatment volume, 36.2 mL; prescribed dose (D95), 36 Gy in 3 fractions; maximum dose, 51.92 Gy; irradiation time, 293 seconds per fraction; and overall treatment duration, five days. IGRT using cone-beam CT (CBCT) was performed immediately before each session to ensure high-precision positioning. To prevent a potential pain flare, oral dexamethasone (4 mg/day) was initiated the day before SBRT. By the fifth day of SBRT (the day of the third fraction for the thigh lesion and the first fraction for the abdominal lesion), the patient's primary complaint of right lower extremity pain had resolved. CBCT scans demonstrated regression of the right thigh lesion. No acute adverse events, including pain flare, were observed.

A non-contrast CT scan performed 10 days after SBRT initiation (the final day of treatment) revealed regression of the right thigh lesion (maximum diameter: 65x60→50x40mm) (Figures 3e, 3f), while the size of the right abdominal lesion remained stable (Figures 4e, 4f). Following SBRT completion, trabectedin therapy was resumed but was suspended due to worsening abdominal pain. A non-contrast CT scan performed seven weeks after SBRT showed further regression of both the right thigh (maximum diameter: 30mmx20mm) (Figures 3g, 3h) and right abdominal lesions (Figures 4g, 4h). However, systemic disease progression was observed outside the irradiated fields, characterized by the enlargement of non-irradiated lesions and the appearance of new lesions.

**Figure 6:**
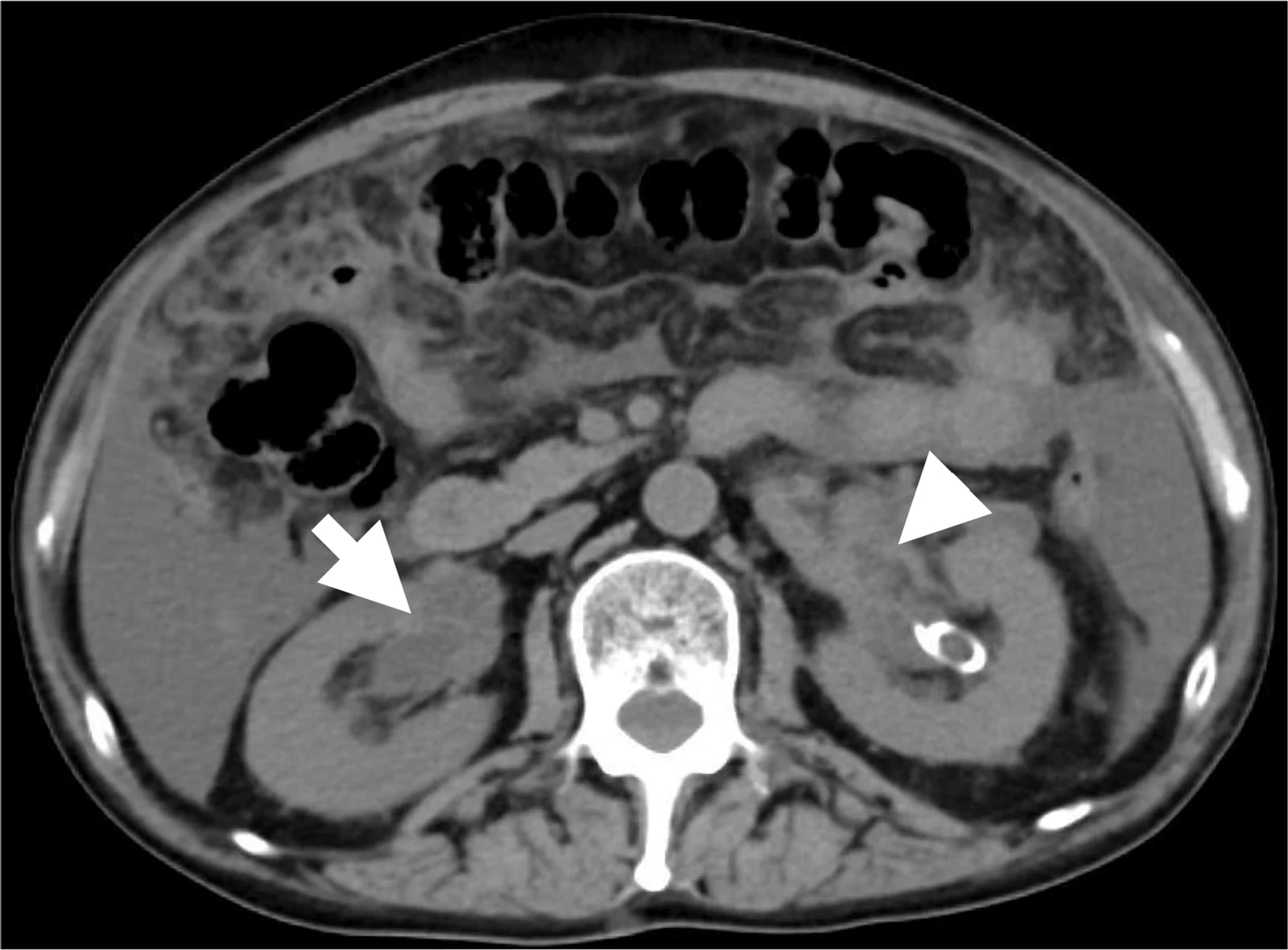
Bilateral renal metastases. Non-contrast CT showing bilateral perirenal lesions after left ureteral stent placement. The white arrow indicates the right lesion, and the white arrowhead indicates the left lesion.

Concurrently, a rapid deterioration in renal function occurred due to intraperitoneal metastases. The estimated glomerular filtration rate (eGFR, mL/min/1.73 m²) was 84.5 (within the normal range) 18 days after SBRT completion but subsequently declined to eGFR 32 at 33 days and to eGFR 26 at 42 days post-treatment. Ureteral stent placement was performed to manage bilateral hydronephrosis caused by the intraperitoneal metastases (Figure 6).

Although additional SBRT was planned and initiated for these intraperitoneal lesions, the rapid, widespread systemic progression could not be controlled.

The patient died of acute renal failure two months after the initial SBRT and 58 months after the primary resection. No recurrence of right lower-limb pain was observed until death, and no SBRT-related adverse events occurred during the clinical course.

## Discussion

In this case, SBRT demonstrated excellent palliative efficacy. The patient experienced pain relief by the fifth day after starting treatment (following two fractions and before the third fraction), and the CCS metastasis shrank rapidly. No acute adverse events were associated with SBRT. Given that the skin of the thigh was compromised by prior surgery, the treatment plan utilized a low marginal dose, a high internal dose, and an every-other-day fractionation schedule [13]. We considered the thigh lesion to be the primary cause of his lower-extremity pain. However, because the abdominal lesion infiltrated the psoas muscle, malignant psoas syndrome [14] could not be ruled out. Therefore, we included the abdominal lesion as a target for SBRT.

CCS is an extremely rare, high-grade sarcoma. In Japan, CCS accounts for 0.52% of all soft tissue sarcomas, with an age-adjusted incidence of 0.024 cases per 100,000 people per year [15]. Conventional radiotherapy and chemotherapy are rarely effective against this tumor [4-6]. Grothues et al. reported the outcomes of local radiotherapy as the initial treatment for 13 cases [4]. Although the literature does not provide details about the irradiation technique, the median radiation dose was 63.2 Gy (range: 50-70 Gy, including a 10-26 Gy boost). In their study, adjuvant radiotherapy failed to salvage cases with poor surgical outcomes. Furthermore, despite high-dose irradiation, recurrence within the irradiation field was observed in two cases.

Radiotherapy is much less invasive than surgery. Therefore, it is a highly viable option for palliative care, especially in elderly or frail patients. X-ray radiotherapy equipment has evolved significantly. SBRT is now feasible on many different models. This modality is highly effective for palliative treatment [7]. It offers precise target definition and superior dose distribution. High-dose fractionated irradiation using SBRT increases the biologically effective dose (BED) and, theoretically, offers an opportunity to overcome the radioresistance of sarcomas to conventional radiotherapy. Traditionally, stereotactic radiotherapy was used primarily for intracranial lesions, such as with Gamma Knife. However, recent technological advancements have expanded its application to extracranial sites, including the neck and trunk. For precise localization, clinicians currently use a variety of methods, including thermoplastic immobilization devices, body immobilization systems, infrared marker systems, and IGRT [10] using CBCT. FFF [8] beams and VMAT [9] significantly reduce the duration of each treatment session. These technologies enable high-precision SBRT in the neck and trunk. SBRT was shown to be a promising treatment for certain patients with metastatic sarcoma [16,17]. Somasundaram et al. [18] reported that SBRT for metastatic sarcoma showed local control rates of 68.6% and 44.5% at one and two years, respectively, even in radioresistant tissues, which is comparable to results in radiosensitive tissues. In particular, they found that sarcoma SBRT had a significant effect on local control when the BED exceeded 95 Gy (α/β=3).

SBRT presents several challenges. Since radiotherapy, including SBRT, is a local therapy, it cannot be expected to function as a systemic therapy. Because a high dose is delivered at once, there is a possibility of serious adverse events due to damage to surrounding healthy tissue. Therefore, high-level quality control is necessary, but these tasks require a great deal of time and effort. In palliative radiation therapy, early initiation of treatment is desirable, but SBRT may take longer to initiate than CRT.

Recently, the usefulness of proton beam therapy and heavy-ion (carbon-ion) radiotherapy for radiation-resistant sarcomas has been reported. These particle beam therapies feature a Bragg peak property, which offers the advantage of suppressing the radiation dose to normal tissues deep within the lesion [19]. However, when a tumor is located immediately beneath the skin, the radiation dose to the skin surface increases. For tumors situated directly under postoperatively fragile skin, as in the present case, the risk of inducing severe skin complications is high, and thus their indication is limited. On the other hand, occasional reports suggest that boron neutron capture therapy (BNCT) may be an effective alternative for CCS [20]. However, even with this treatment, serious side effects, such as mucosal ulcers, perforations, and fistulas caused by necrosis at the irradiation site, have been reported. Therefore, BNCT is not necessarily an appropriate treatment for lesions located directly under fragile postoperative skin, as in this case.

Currently, few reports exist on the use of SBRT for CCS. Due to the rarity of this disease, conducting large-scale prospective studies is highly challenging; therefore, accumulating and analyzing data from individual cases remains essential. Although follow-up periods in our case have been short, and long-term local control rates and late adverse events remain poorly understood, SBRT may represent a viable palliative treatment option for CCS.

## Conclusions

Surgical resection is the primary treatment modality for CCS because it was traditionally considered highly resistant to radiotherapy and chemotherapy. However, this case report demonstrates that SBRT may serve as a viable palliative option to reduce symptom burden and improve quality of life, even for CCS. Furthermore, advancements in radiotherapy systems are driving the development of SBRT. Currently, data regarding long-term local control and late adverse events remain limited. Therefore, further case reports, studies, and longer follow-up periods are required to confirm these findings.

## References

[1] Cornillie J, van Cann T, Wozniak A, Hompes D, Schöffski P (2016). Biology and management of clear cell sarcoma: state of the art and future perspectives. Expert Rev Anticancer Ther.

[2] Kawai A, Hosono A, Nakayama R (2007). Clear cell sarcoma of tendons and aponeuroses: a study of 75 patients. Cancer.

[3] Gonzaga MI, Grant L, Curtin C, Gootee J, Silberstein P, Voth E (2018). The epidemiology and survivorship of clear cell sarcoma: a National Cancer Database (NCDB) review. J Cancer Res Clin Oncol.

[4] Grothues J, Hardes J, Agaimy A (2024). Prognostic factors in clear cell sarcoma: an analysis of soft tissue sarcoma in 43 cases. J Cancer Res Clin Oncol.

[5] Ikuta K, Nishida Y, Imagama S, Tanaka K, Ozaki T (2023). The current management of clear cell sarcoma. Jpn J Clin Oncol.

[6] Jones RL, Constantinidou A, Thway K (2011). Chemotherapy in clear cell sarcoma. Med Oncol.

[7] Sahgal A, Myrehaug SD, Siva S (2021). Stereotactic body radiotherapy versus conventional external beam radiotherapy in patients with painful spinal metastases: an open-label, multicentre, randomised, controlled, phase 2/3 trial. Lancet Oncol.

[8] Prendergast BM, Fiveash JB, Popple RA (2013). Flattening filter-free linac improves treatment delivery efficiency in stereotactic body radiation therapy. J Appl Clin Med Phys.

[9] Teoh M, Clark CH, Wood K, Whitaker S, Nisbet A (2011). Volumetric modulated arc therapy: a review of current literature and clinical use in practice. Br J Radiol.

[10] Dawson LA, Sharpe MB (2006). Image-guided radiotherapy: rationale, benefits, and limitations. Lancet Oncol.

[11] Bindels BJ, Mercier C, Gal R (2024). Stereotactic body and conventional radiotherapy for painful bone metastases: a systematic review and meta-analysis. JAMA Netw Open.

[12] Kawata K, Kishigami Y, Hirashima H (2025). Performance evaluation of a second-generation O-ring-shaped image-guided radiotherapy system with a gimbal-mounted linear accelerator and real-time tracking capabilities. J Appl Clin Med Phys.

[13] Yazici G, Sanlı TY, Cengiz M (2013). A simple strategy to decrease fatal carotid blowout syndrome after stereotactic body reirradiaton for recurrent head and neck cancers. Radiat Oncol.

[14] Suraj D, Zhang A, Appelbaum T (2023). Clinical presentation and management of malignant psoas syndrome: a scoping review of case reports and case series. Cureus.

[15] Takemori T, Ogura K, Morizane C (2024). Clear cell sarcoma in Japan: an analysis of the population-based cancer registry in Japan. Jpn J Clin Oncol.

[16] Elledge CR, Krasin MJ, Ladra MM (2021). A multi-institutional phase 2 trial of stereotactic body radiotherapy in the treatment of bone metastases in pediatric and young adult patients with sarcoma. Cancer.

[17] Parsai S, Sedor G, Smile TD (2021). Multiple site SBRT in pediatric, adolescent, and young adult patients with recurrent and/or metastatic sarcoma. Am J Clin Oncol.

[18] Somasundaram E, D Smile T, Halima A (2022). Association between biologically effective dose and local control after stereotactic body radiotherapy for metastatic sarcoma. J Radiosurg SBRT.

[19] Webster M, Podgorsak A, Li F (2025). New approaches in radiotherapy. Cancers (Basel).

[20] Fujimoto T, Suzuki M, Sudo T (2020). Boron neutron capture therapy for clear cell sarcoma. Appl Radiat Isot.

